# Initial experience with augmented reality in planning renal access for PCNL

**DOI:** 10.1007/s00240-025-01730-3

**Published:** 2025-04-09

**Authors:** Rinat Lasmanovich, Maayan Dagan, Orel Hemo, Shai Tejman-Yarden, Dorit E. Zilberman, Oliana Vazhgovsky, Zohar A. Dotan, Nir Kleinmann, Asaf Shvero

**Affiliations:** 1 Department of Urology, Sheba Medical Center, Ramat Gan, Israel; 2 The Engineering Medical Research Lab, Sheba Medical Center, Ramat Gan, Israel; 3 Faculty of Medicine, Tel Aviv University, Tel Aviv, Israel

**Keywords:** PCNL, Percutaneous nephrolithotomy, 3D, Three-dimensional, AR, Augmented reality

## Abstract

Achieving renal access during percutaneous nephrolithotomy is challenging, with a complication rate of 10–20%. This study aims to assess a novel technique for planning access to the renal collecting system using augmented reality (AR). Using Digital Imaging and Communications in Medicine software (DICOM 2-print), we generated 3-dimensional (3D) models of patients with different types of kidney stones (staghorn, lower pole, and pelvic stones) who underwent PCNL between 2018 and 2022. After viewing the patient’s CT scans, surgeons explored the anatomical models using an AR system with a stereoscopic 3D lens array display. Using questionnaires, we quantitatively estimated the model’s contribution on a scale of “1” (“poor”) to “5” (“excellent”) to the surgeon’s understanding of stone location, patient anatomy, and ease of access compared to 2D CT imaging. A total of 38 questionnaires were completed by 13 urologists. Estimating renal location, renal pelvis, and stone mass were better demonstrated by AR (5 vs. 4, *p* < 0.001). The orientation of adjacent organs was better understood using AR (5 vs. 4, *p* = 0.004). In 86.8% of cases, surgeons preferred using AR models both before and during surgery, with 69.2% suggesting that AR could enhance procedural safety. The time frames for testing the models differed between senior and junior practitioners (*p* = 0.016) and were significantly reduced with model experience (*p* < 0.001). When comparing CT to 3D imaging, AR models provide a better understating of stone location, patient anatomy, and route of access to the collecting system. Further research is required to implement this innovative technique preoperatively and intra-operatively.

**Supplementary Information:**

The online version contains supplementary material available at 10.1007/s00240-025-01730-3.

## Introduction

Percutaneous nephrolithotomy (PCNL) is a common guideline-endorsed technique and is the first-line treatment for kidney stones larger than 2-cm and Staghorn calculi [1–4]. For 1–2 cm renal stones, it is frequently used when they are in the kidney’s lower pole. For stones smaller than 1 cm, PCNL is typically reserved for cases where ESWL or URS failed, or in the presence of acute infundibulo-pelvic angles or calyceal diverticula [2–4, 6] As the most efficient means to remove large kidney stones, PCNL has achieved stone-clearance rates as high as 90% [3, 5].

Despite its high stone-free rate and the improvements in instruments (i.e., flexible ureteroscopes) as well as lithotripsy technology (i.e., ultrasound/pneumatic devices and lasers), its overall complication rate is relatively high, ranges between 10% and 20%. Most contemporary series show bleeding requiring transfusion in 5-10% of cases, thoracic complications or adjacent organ injury in 3% of cases, severe sepsis or delayed bleeding requiring angio-embolization up to 1%, and death up to 0.3% [3, 5]. Additionally, extended procedure time may expose patients and operational team members to ionizing radiation and increase anesthesia complications [7, 8].

The most important yet challenging part of a successful PCNL is achieving suitable access to the renal collecting system [9–11]. Currently, medical centers utilize 2-dimensional (2D) imaging tools (i.e., CT scans, ultrasound devices, and fluoroscopy) to plan renal access. Nevertheless, the necessity for a more accurate preoperative planning tool is clear, considering the procedure’s prolonged operating time and high complication rates.

Numerous novel techniques were offered to meet this need. The real-time awareness of the position and orientation of the target kidney has led to the development of 3D models; a recent systemic review found that using 3D printing models prior to nephrolithiasis surgery resulted in significant advantages in terms of operation time, stone clearance rates, puncture accuracy, hospital stay, blood loss, and the incidence of complications [12]. Computer algorithms were designed to process CT scans using segmentation and reconstruction techniques, enhancing 3D images to better demonstrate patient’s anatomy through augmented reality (AR) and virtual reality (VR) technologies [9, 11]. Consequently, 3D models proved to be beneficial for training and surgery planning purposes [9, 11, 13, 14]. For PCNL, 3D and AR models were first tested on ex-vivo models showing their potential to ensure a correct angle and an access point [10]. As this field continues to expand, a limited number of studies have been conducted intraoperatively, yet they have positively impacted decision-making and demonstrated promising results [9, 15, 16, 20, 21].

Given the potential impact of these innovative technologies, our study aimed to further assess the preoperative utility of AR among urologists, focusing on its role in enhancing their understanding of patient anatomy, particularly regarding renal collecting system access and stone localization.

## Materials and methods

The study was performed under the ethical standards of the declaration of Helsinki and its later amendments. The protocol was approved by the local ethics committee at The Sheba Medical Center (approval 7611-20-SMC). Data was treated according to the principles of good clinical practice (GCP).

We retrospectively reviewed the medical records of patients who underwent PCNL during 2018–2022 at our institution. We then selected three cases that demonstrated three types of renal stone: The first subject had a right-sided staghorn stone, the second had two stones in the left kidney - a 6-mm stone located at the lower pole, and a 4-cm stone in the renal pelvis. The third subject had a 3-cm stone in the pelvis of the left kidney. The second subject had a nephrostomy tube before the surgery. Patients had no anatomical deformations or malformation of kidneys. Clinical information was available to the surgeons. Subjects’ characteristics are summarized in Table 1.

**Table 1:** Subjects’ characteristics

	Gender	Age	Medical background	Side	Type of stone, cm	Stone density (Hu)	Length of surgery, min	Days hospitalized	Complications
Patient#1	F	65	DM, HTN, Dyslipidemia	Right	Staghorn stone	779.23	138	4	None
Patient#2	M	49	DCM, CKD, HTN, gout, OSA	Right	Lower pole, 0.6 cm & pelvic stone, 4 cm	1320	232	6	Post-operative infection
Patient#3	M	65	Healthy	Left	Pelvic stone, 3 cm	1695.9	208	3	None

F; female, M; male, DM; diabetes mellitus, HTN; hypertension, DCM; dilated cardiomyopathy, CKD; chronic kidney disease, OSA; obstructive sleep apnea

All urologists (22 in total) in our tertiary center’s department were invited to explore the AR model. The participant selection was based on this open invitation, resulting in a 60% response rate.

### Augmented reality imaging protocol

**Fig. 1:**
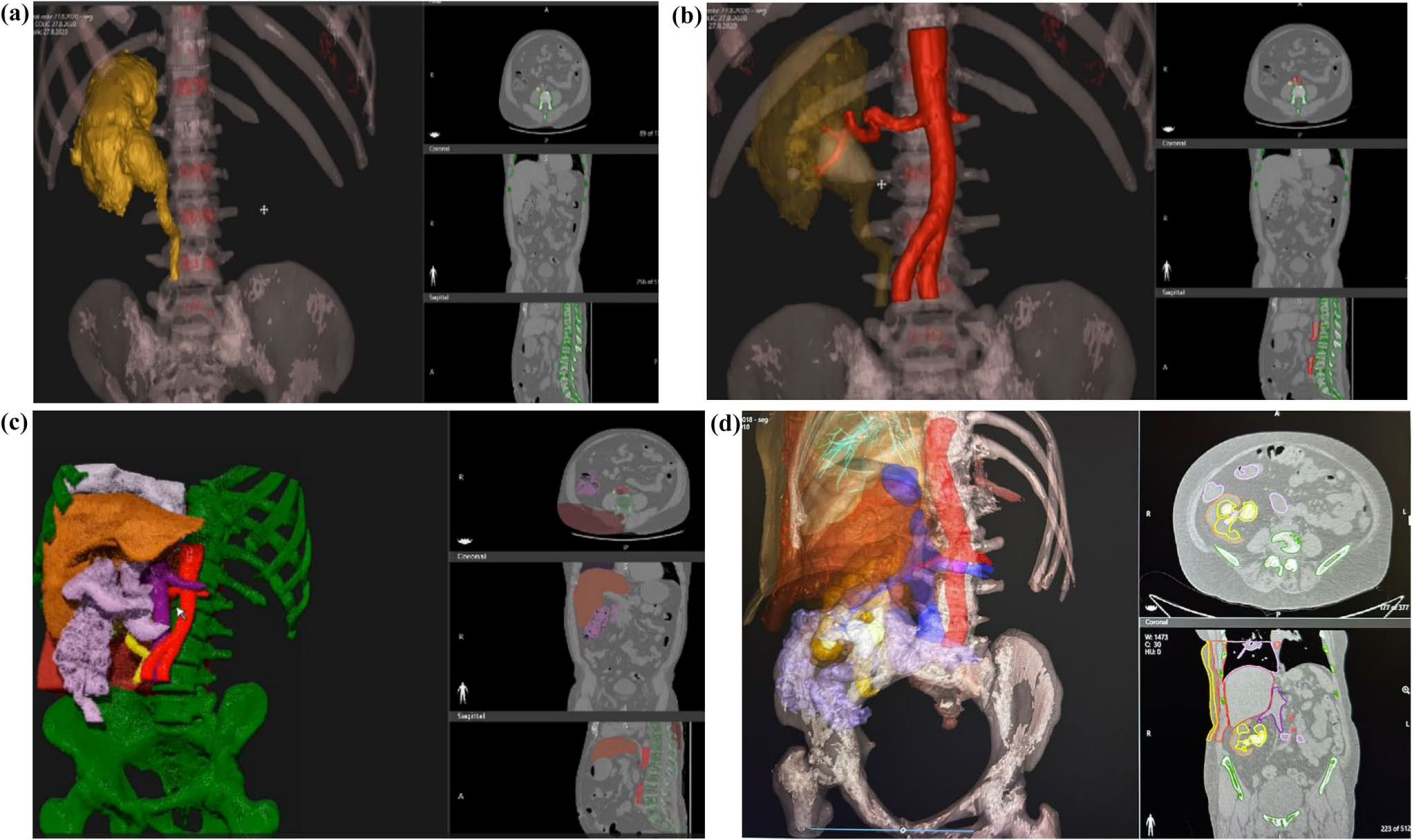
The use of axial, coronal and sagittal CT planes were applied throughout the construction of the model. The relationship between the 2D model (from the right) and the 3D model (from the left) can be observed. Each picture demonstrates a different opacity of the presented organs. **a** The construction of the skeletal framework and the positioning of the kidney, renal pelvis and proximal ureter in space is seen. **b** Next, calculating the location of the stone in the renal pelvis (painted in white), and the positioning of blood vessels is seen (aorta, renal artery and branches). Note, the construction of the IVC, renal vein and branches is not demonstrated. **c** The positioning of adjacent organs to the right kidney; the liver (in brown), intestines (hepatic flexure, in purple), IVC (dark purple), aorta (red), right lung (in purple), and ureter (in yellow). **d** A model for a right staghorn stone. The staghorn stone painted white, and the collecting system painted yellow. In addition, soft tissues, including muscles and skin are also presented. CT: computed tomography, 2D: two-dimensional, 3D: three-dimensional IVC; inferior vena cava

For each patient, the CT scan was loaded as Digital Imaging and Communications in Medicine (DICOM) file on the DICOM-to-Print (D2P) software (3D Systems Inc., Littleton, CO), for tissue segmentation and 3D model preparation. The software can be installed on any licensed desktop or laptop. Images segmentation was performed in medical engineering lab, by a certified technician and focused on all aspects of relevant structures including renal stones, the collecting system, renal parenchyma, ipsilateral renal blood vessels, ipsilateral adjacent organs (liver, spleen intestine, lungs), skeletal structures (ribs, spine, pelvic bones), and the patient’s skin. Images are demonstrated in Fig. 1.

The models were created with the guidance of an endo-urologist expert. The average time to create a 3D model was 4.5 h.

**Fig. 2:**
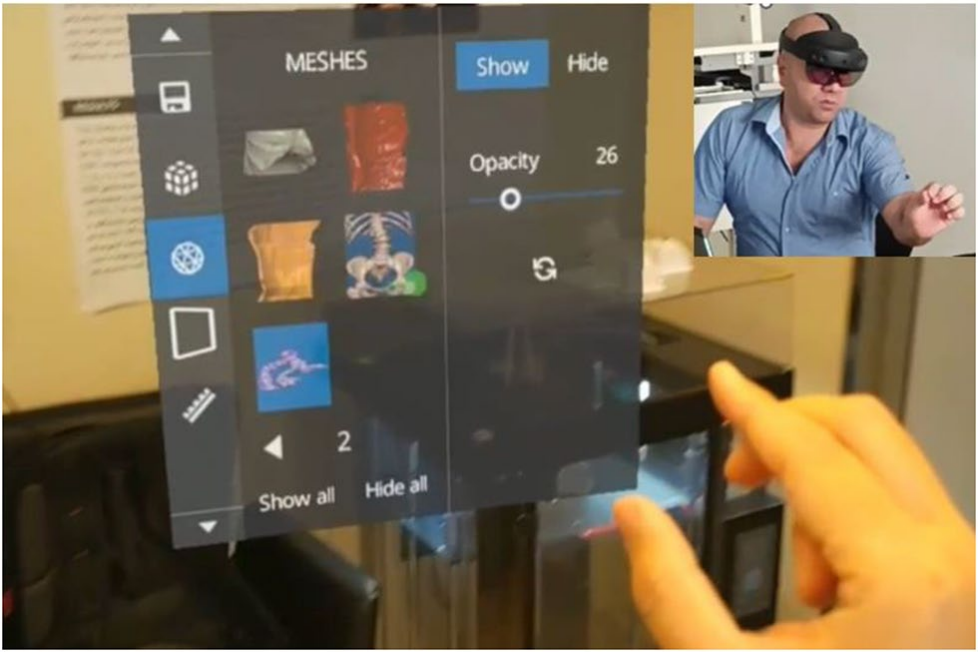
In the small upper-right picture, the urologist is wearing HoloLens glasses, which project an array display. This display enables adjustments to the 3D model, such as opacity, size, and shape. This functionality allows urologists to interact with the model by highlighting specific organs and visualizing the stone along with its surrounding anatomy in detail

For AR visualization, a wireless HoloLens First Generation (HL1) Head Mounted Unit (HMU) was worn by the urologist (Microsoft Corporation, Redmond, WA) [17–19]. Applying the D2P software, AR system-based anatomical navigation was achieved using a stereoscopic 3D HoloLens lens composed of sensors, camera, displays and see-through holographic lenses (waveguides) as well as second-generation custom-built holographic processing unit, audio and speech systems. With 3D glasses, the models appeared in front of the urologist. Using real-time hand and eye tracking, the urologist could move through, rotate, adjust opacity, and enlarge anatomical structures (renal stone, kidney, and adjacent organs) using hand movements and head positioning. These interactions were possible while sitting, standing, or leaning within the virtual space, as demonstrated in Fig. 2.

Videos demonstrating models’ development as well as display demonstration as seen through HoloLens 3D glasses are provided as *supplementary information.*

### The questionnaires protocol

For each patient, each surgeon was initially provided with a non-contrast CT scan, which they could examine in axial, sagittal, and coronal views as needed for an unlimited duration. The surgeons were then tasked with planning percutaneous access to the collecting system. Then, the surgeon went through a short training session for 10–15 min with a lab technician on the AR platform. The surgeon was then presented with an AR model corresponding to the previously viewed CT scan, using wireless HoloLens glasses. The surgeon had unlimited time to explore the anatomical display and to plan the percutaneous access. In the subsequent step, the surgeon was requested to complete an 8-question questionnaire designed to quantitatively assess the advantages of the AR model, with responses rated on a scale from 1 to 5; The first 5 questions assessed the understanding of the patient’s specific anatomy including kidney and collecting system position, adjacent organs, and their relation to the kidney, stone characteristics, and lastly - renal access (“1” being “to a very low degree” to “5” being “to a very high degree”). Additional 3 questions evaluated the potential contribution of the AR model before and during the surgery and its potential impact on the procedure safety through the urologist’s perception (“1” being “Disagree” to “5” being “Strongly agree”). The questionnaire is provided in the *Supplementary information.*

### Statistical analysis

Continuous variables are presented as median and IQR (25th, 75th percentile). The Mann-Whitney U test was used for continuous variables and the Kruskal-Wallis test for comparing multiple abnormal distributed continuous variables. Categorical variables are presented as counts(percentages). Friedman’s and Chi-square tests for abnormal distribution were used to compare the AR model to CT scans and to estimate the AR model as a stand-alone tool. Statistical analyses were performed using IBM SPSS Statistics 29 (IBM Corp, Armonk, New York). The level of statistical significance was set at *P* < 0.05.

## Results

3D models of renal stones were successfully segmented and constructed for all three patients by a lab technician. All relevant anatomical structures as mentioned above were constructed and were visible. The median age of patients was 65 years (IQR: 61 − 57) with a median stone density of 1,320 Hounsfield units (IQR: 1184.8-1049.62). One patient had a post-operative infection, which was resolved with antibiotic use.

A total of 38 questionnaires were completed by 13 urologists of them 6 seniors and 7 juniors. Demographic data of the participating surgeons is given in Table 2.

**Table 2:** Demographic data of the participating surgeons

Participant no.*	Age	Position	Years of experience	Main specialty	No. of PCNL performed
1	48	S	19	Endourology	> 100
2	44	S	14	Endourology & BPH	> 100
3	45	S	15	Functional Urology	> 100
4	40	S	8	Oncology-Urology &BPH	< 50
5	68	S	39	Andrology & BPH	50–100
6	52	S	17	Oncology-Urology &BPH	50–100
7	34	R	2	-	< 50
8	36	R	4	-	< 50
9	32	R	5	-	< 50
10	34	R	6	-	< 50
11	31	R	3	-	< 50
12	31	R	3	-	< 50
13	36	R	4	-	< 50

*Urology surgeon, R: Resident, S: Senior, BPH: Benign prostatic hyperplasia

**Fig. 3:**
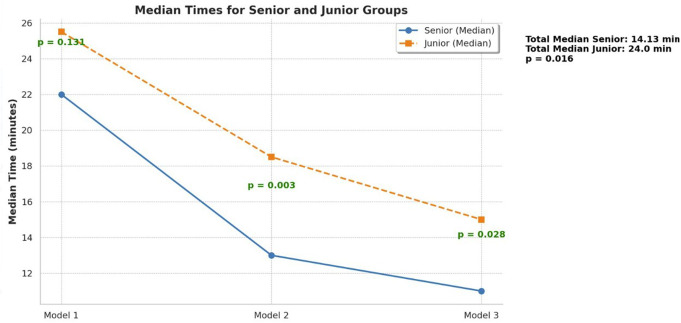
Median times for senior and junior groups. Senior results are shown with a solid blue line (circles), and Junior results with a dashed orange line (squares). The x-axis represents the three models, with p-values in green indicating statistical significance. The overall median times (top right) confirm that seniors completed tasks significantly faster, especially in Models 2 and 3

The median time dedicated to reviewing CT scans and for AR model exploration per patient was 18 min (IQR: 23.75–13.25). When comparing between senior and junior time frames, median time was 14.13 and 23 min per patient, respectively (CI 95%, *P* = 0.016). The average time that was required for participants to experiment with AR models was significantly reduced over time 30.42, 17.49, 9.63 min for patients 1, 2 and 3, respectively (*P* < 0.001). Comparison of median times for seniors and juniors are demonstrated in Fig. 3.

**Fig. 4:**
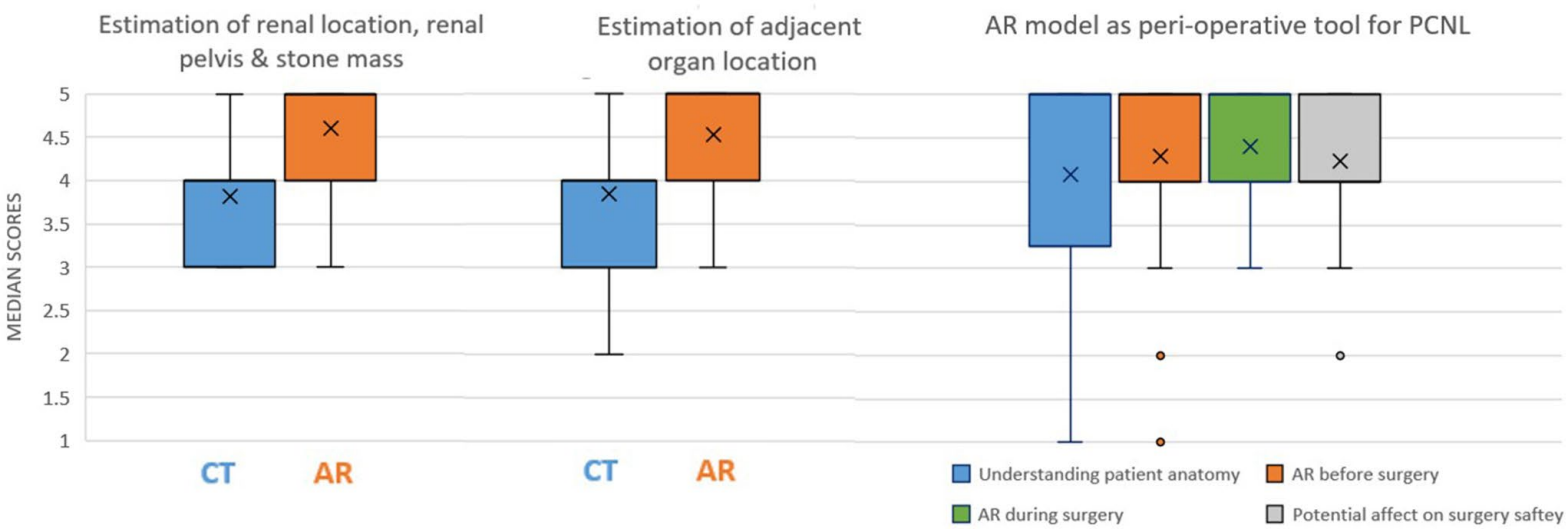
Box plots from right to left: Surgeons’ ratings for CT vs. AR are presented. The rightmost plot compares kidney location, renal pelvis, and stone evaluation (**CT: 4 (4–3)**,** AR: 5 (5–4)**). The middle plot presents adjacent organ location ratings (**CT: 4 (4–3)**,** AR: 5 (5–4)**). The leftmost plot assesses the AR model as a peri-operative tool for PCNL, including its contribution via **3D glasses** to anatomical perception **[5 (5–3.25)]**, its desirability **before [5 (5–4)]** and **during [5 (5–4)]** surgery, and its potential impact on procedure safety **[4 (5–4)]**

Estimation of renal location, renal pelvis, and stone mass was better demonstrated by the AR models compared to CT (5 vs. 4, *p* < 0.001). In addition, understanding the relation of the kidney to the adjacent organs was also greater using the AR model (5 vs. 4, *p* = 0.004). In 86.8% of cases, surgeons stated they would prefer to have the AR model before and during the surgery (defined as “3” and above); moreover, in 21 out of 35 questionnaires (60%) the urologist marked “5” (i.e., strongly agree) when asked if they would like to use AR model before surgery, and in 20 out of 38 questionnaires (52.6%) the urologists marked “5” when were asked if they would want to use the AR model during the procedure. When asked about the potential contribution to procedure safety (defined as “3” and above), 69.2% of surgeons thought that AR could increase its safety. Boxplot summarizing results are shown in Fig. 4.

Functionalities and applications of AR system compared to CT scan are given in Table 3.

**Table 3:** Functionalities, applications and costs of AR system compared to CT scan

Feature	Augmented Reality (AR)	Non-contrast CT scan
Visualization	Provides real-time, 3D overlay of patient anatomy onto the surgical field, enabling precise guidance.	Offers high-resolution 2D images of kidney structures preoperatively.
	Can be modified and translated into several positions for different orientation (interactive).	Static images demonstrate a single position on a computer screen.
	Improves spatial perception	
Radiation Exposure	None.	Approximate effective radiation dose of 10 mSv^ per abdomen and pelvis scan*
	May decrease surgery time and repeated fluoroscopy during PCNL (1-100 mSv depends on surgery course) **	
Preoperative Planning	Integrates with 2D imaging data (e.g., CT, MRI) to create interactive surgical maps.	Provides detailed anatomical data for planning, without interactive elements.
	Engineering model planning is needed (Lab technician)	Requires technician, radiologist.
Accuracy	- Holographic resolution 2k 3:2 light engines - Holographic density > 2.5k radiants (light points/radian).	Field of view: 350 mm (should be adjusted to increase in-plane resolution).
	Enhances the usability and interactivity of data.	Provides higher native resolution (sub-millimeter).
	Imaging is projected in front of the surgeon, providing hand-free access to critical data.	Requires surgeon’s interpretation and memory reconstruction.
Learning Curve	Requires a short training	Standard imaging technique, widely understood by urologists.
	Can be trained in an office setting (patient-specific model)	
	Enhance surgical skill acquisition	
Special features and applications	Color-coded data displays critical structures, such as blood vessels and nerves.	All organs, including blood vessels, appear in greyscale. Nerves are not usually visible
	Allows direct interaction with patients and environment.	Working in front of a computer screen
	Improves doctor-patient communication by providing interactive visual explanations of procedures.	There is no doctor-patient interaction.

AR; Augmented reality, 3D; 3-dimentionalm, 2D; 2-dimentional, DICOM: Digital imaging and communication in medicine, IVC; Intra venous urography, MRI; Magnetic resonance imaging

^ The radiation dose, a measure of ionizing energy absorbed per unit of mass, is expressed in grays (Gy) or milligrays (mGy); 1 Gy = 1 J per kilogram. The radiation dose is often expressed as an equivalent dose in sieverts (Sv) or millisieverts (mSv). For x-ray radiation, which is the type used in CT scanners, 1 mSv = 1 mGy

* Brenner, D. J., & Hall, E. J. (2007). Computed Tomography — An Increasing Source of Radiation Exposure. New England Journal of Medicine, 357(22), 2277–2284

** Elmarakbi, A.A., Elnoiry, A.K., Deiab, N. et al. (2024) Comparative study between radiation exposure in common urological procedures. *Beni-Suef Univ J Basic Appl Sci* 13, 2

## Discussion

In the last decade, advancements in 3D illustration technology have offered solutions to reduce PCNL complication rates by reflecting patient anatomy [12, 15–19]. VR and AR imaging demonstrated perioperative efficiency, serving as both a training tool and a method for renal access planning [11–13, 16]. Rassweiler-Seyfried et al. further tested that idea on iPad-assisted marker-based navigation to visualize organ boundaries using AR and 3D models before PCNL. Examining radiation exposure showed a significant difference in favor of the standard puncturing method and puncture time. However, there was no significant difference in puncturing attempts [16]. Parkhomenko et al. used CT-based immersive-VR (iVR) models that immerse the observer in an interactive 3D simulation of the patient’s anatomy. They found that iVR improved urologists’ understanding of the renal anatomy which altered the operative approach in 40% of cases. Retrospectively, iVR had the benefits of decreased fluoroscopy time and less blood loss [14]. We found one study that tested 3D MRI based models using HoloLens glasses during PCNL. Porpiglia et al. revealed a significantly shorter radiation exposure time for the 3D MRI group even though the puncture time was longer in comparison to the controls. It also led to a higher success rate for renal puncture at the first attempt [15]. In the current study, we combined both AR models and 3D glasses to create a more realistic way to explore patients’ anatomy, and to contribute to the scientific literature by validating its advantages before applying it in the operating room (OR).

Learning with 3D models enhances mental model development, improves anatomic education, and benefits student performance while reducing cognitive load, ultimately leading to better diagnosis and treatment [23–25]. Hence, we believe the AR model is expected to serve as a valuable tool for practice and learning specifically prior to PCNL, considering its complex surgical requirements.

Based on this study results, the AR model contribution can be explained by the process of cognitive image translation and the significance of element depth perception. With the absence of depth cues, perception is not reliable [24]. Such cues include the following: linear perspective (parallel lines converge at a distant point on the horizon), relative size (occlusion of an object by a proximal one affects its size), shading and lighting (e.g., cast by an object), texture gradient (surface features on objects) and prior knowledge. The absence of most elements, as seen in a 2D CT scan, can introduce bias in how surgeons perceive organ distances and anatomical structures [23, 26]. This missing information impacts surgical performance and outcomes, as demonstrated in robotic partial nephrectomy [27, 28]. AR offers more comprehensive depth cues thus, in our view, incorporating it could impact surgical planning decisions, enhancing surgeon’s orientation and navigation abilities while reducing the likelihood of adverse events.

While 3D printing is a well-established technique for PCNL [9–13], the use of AR models for intraoperative applications is gaining momentum [9, 14–16, 20, 21]. Ferraguti et al. demonstrated that integrating AR with robotic assistance can improve surgical outcomes in both pre- and intraoperative phases although the study was conducted as a matched-pair analysis on a sample of 11 users [21]. Although large RCTs and prospective PCNL studies using AR are lacking, AR has been favored among urologists for intraoperative guidance and training, particularly in prostate and kidney cancer surgeries. Among available technologies, HoloLens was perceived as the most effective for surgical planning [29].

The degree of experience is a well-known factor in a urologist’s performance. Our study shows that learning to manipulate the AR model requires a reasonable amount of time. Furthermore, the time needed for planning access, based on modeled renal collecting systems, decreased as urologists progressed from model to model, indicating a fast-learning curve. In our opinion, the availability of these models in laboratory settings enables training and provides unrestricted practice time, which results in more effective surgical preparation based on patient-specific models. However, a deeper understanding of AR’s impact on the urologist’s learning curve is highly valuable and warrants a large-scale prospective study.

We anticipate several challenges in using AR in clinical settings. Financially, software and specialized equipment costs vary widely, ranging from hundreds to thousands of dollars, depending on factors such as software complexity, customization level, security measures, integration capabilities, and more. Software licenses are usually annual, whereas equipment may be a one-time investment. Pre-operative, the AR models are generated from CT scans, hence a low-quality scan may produce lower-quality models. Additionally, all patients were scanned in the supine position, which does not align with surgical positioning or account for breathing-related shifts in organ location. Solutions could include scanning patients in surgery-like positions (e.g., supine or prone with flank padding), using skin-based markers or skeletal landmarks (e.g., ribs, ASIS, etc.), and synchronizing the breathing cycle of the CT and the actual puncture.

While incorporating this state-of-the-art technology into the OR, challenges including ensuring the technology complies with medical regulatory bodies (e.g., FDA, CE marking, HIPAA for data security), conduct risk assessments and meet hospital accreditation and safety standards may rise. Furthermore, encourage collaboration among OR staff, anesthesiologists, surgeons, nurses and lab technicians in adopting new equipment and technology, as well as in establishing a clinical workflow, which may initially extend operating time. We do not anticipate any issues with OR space, electrical, networking, or other infrastructure needs, as the AR system is highly portable (requiring only 3D glasses and a laptop, as described earlier).

The major limitation of this study is the subjective nature of the data collected through self-assessed questionnaires. Second, the benefit of the AR models was not assessed intra-operatively (i.e., puncture location, puncture success rates, operative time, postoperative complications). This weakens the study findings. Third, the study includes a relatively small number of patients. And lastly, the urologists who participated in the study have different levels of experience in PCNL procedures, which may influence their model perception and generalizability of the study. Nevertheless, more than a dozen urologists evaluated the models, which is comparable to other published studies [14, 16, 21, 22].

Despite these limitations, we believe this study contributes to the development and assessment of AR models using 3D glasses as a tool for PCNL preoperative planning, adding to the literature in this expanding field. Herein, we developed and evaluated the benefits of such a model from a urologist’s perspective before its intraoperative application. Based on our findings, further studies should assess its use by incorporating objective metrics during surgery. We emphasize that this product remains investigational and is not yet approved for clinical use.

## Conclusions

In the pre-operative setting for planning renal collecting system access during PCNL, the use of AR displayed through stereoscopic 3D glasses offers several advantages over 2D imaging. It provides a clearer demonstration of stone location, patient anatomy, and ease of access to the renal collecting system. These benefits were emphasized by urologists who used the AR models developed under the guidance of endourologists and based on patients’ CT scans.

As a result, preoperative review of AR models using 3D glasses has the potential to increase procedure safety. Prospective, large-scale studies to examine this model intra-operatively are required to implement and assess this state-of-the-art technique.

## Electronic supplementary material

Below is the link to the electronic supplementary material.

Supplementary Material 1

Supplementary Material 2

Supplementary Material 3

## Data Availability

No datasets were generated or analysed during the current study.
