# Supplementary material for: Initial experience with augmented reality in planning renal access for PCNL
Source: Urolithiasis. 2025 Apr 9;53(1):69. doi: 10.1007/s00240-025-01730-3 (PMC11982134; doi:10.1007/s00240-025-01730-3)
Supplement: Supplementary file 3 — Supplementary Material 3 [file 240_2025_1730_MOESM3_ESM.pdf]

Initial experience with Augmented Reality models for planning PCNL

Only those who reviewed the patient's CT before testing the AR model

Please circle the relevant answer

Patient: 1 2 3

Age: ________________

Position: Resident Senior urologist

Years of experience: _______________

Main specialty: ___________________

No. of PCNL performed: <50 50-100 100<

Estimation / *Questions no.*	*1* *To a very low degree*	*2* *To a low degree*	*3* *Moderately*	*4* *To a high degree)*	*5* *To a very high degree*
1.To what degree could you estimate the spatial positioning of the kidney, the collecting system, and the stone mass based on axial, sagittal, and coronal CT sections?					
2.To what degree could you estimate the spatial positioning of the kidney, the collecting system, and the stone mass based on the AR model?					
3.To what degree could you estimate the location of the adjacent organs based on CT?					
4.To what degree could you estimate the location of the adjacent organs based on the AR; model?					

5.To what degree did the AR model assist you in understanding the patient's anatomical structure?					
	1<br>*Disagree*	*2*<br>*Somewhat disagree*	*3*<br>*Moderately agree*	*4*<br>*Somewhat agree*	*5*<br>*Strongly agree*
6.I would prefer to see the AR model display before the procedure (pre-operatively).					
7.I would like to view the AR model display during the procedure (intra-operatively).					
8.By using the AR model, I expect safe access with minimal complications to the renal pelvis, and the collecting system compared to percutaneous access based solely on the CT scans.					
